# Jujuboside B Reverse CUMS-Promoted Tumor Progression via Blocking PI3K/Akt and MAPK/ERK and Dephosphorylating CREB Signaling

**DOI:** 10.1155/2022/5211368

**Published:** 2022-10-08

**Authors:** Zhen Yang, Weijia Cai, Yitian Chen, Zhijun Guo, Zhijun Xiao, Ting Zhou, Yuanchi Cheng, Feng Xu

**Affiliations:** ^1^Fengxian Hospital, Southern Medical University, Shanghai 201499, China; ^2^School of Pharmaceutical Sciences, Southern Medical University, Guangzhou 510515, China; ^3^Sixth People's Hospital South Campus, Shanghai Jiao Tong University, Shanghai 201499, China

## Abstract

**Background:**

Jujuboside B (JUB) is a saponins isolated from the seeds of *Zizyphi jujuba var. spinosi*, which is used to treat mental illness and is reported recently to induce cancer cell apoptosis. As our previous research showed chronic stress promoted tumor growth, this work aims to investigate whether JUB exert antitumor effect in addition to its antidepressant effect and possible mechanism.

**Methods:**

56 female C57BL/6 mice were grouped into 7 groups: A (blank control), B (tumor-bearing control), C (tumor-bearing + JUB), D (CUMS control), E (CUMS + JUB), F (tumor-bearing + CUMS), and G (tumor-bearing + CUMS + JUB). Groups C, E, G, B, D, and F were administered, respectively, with JUB (40 mg/kg/day) or vehicle for 2 weeks. Serum 5-HT, Trp (tryptophane), inflammatory cytokines TNF-*α*, IL-4, -6, and -10 levels were detected by ELISA. The tumors in groups B and F were isolated for RNA-seq sequencing. Protein and mRNA expression of Bax, Bcl-2, p-PI3K, p-Akt, p-MAPK, p-ERK, and p-CREB in tumor tissues were detected. In vitro, A549 cells were stimulated with JUB (60 *μ*mol/L), in which proliferation rate and colony formation rate were detected. The PI3K/Akt and, MAPK/ERK pathway were measured.

**Results:**

Chronic stress successfully induced the depression-like phenotype (group D vs. A) and promoted tumor growth (group B vs. F). JUB significantly ameliorated the depression-like phenotype and increased 5-HT, Trp levels (group D vs. E), and reversing CUMS-induced tumor progression. Meanwhile, JUB decreased inflammatory cytokine levels. Chronic stress upregulated the phosphorylation levels of PI3K/Akt/MAPK/ERK/CREB; JUB reversed this regulation. JUB significantly inhibited cell viability, colony formation rate, and downregulated the phosphorylation levels of PI3K/Akt/MAPK/ERK/CREB in vitro.

**Conclusions:**

JUB reverses CUMS-promoted tumor progression in tumor-bearing mice with depression-like phenotype. JUB exerts the dual beneficial effect on tumor growth and depression-like phenotype by blocking the signal transduction pathway of PI3K/Akt, MAPK/ERK, and dephosphorylating the downstream signaling regulator CREB.

## 1. Introduction

Jujuboside B (JUB) is a saponin isolated from the seeds of *Zizyphi jujuba var. spinosi*, which is used to treat mental illness, neurodegenerative diseases, and so on. It is reported to possess multiple pharmacological activities. Recently, JUB is found to induce cancer cell apoptosis and exert anticancer activity in breast and colon cancer [1, 2].

Depression is a common comorbidity in cancer patients. In our previous work, we noted that about 30% of cancer patients occurred mild to major depression [3, 4]. Many factors lead to comorbid depression in cancer patients. First of all, being confirmed as malignancy is a great negative life event for most people. Second, the side effects and toxicities of chemotherapy/radiotherapy are often severe and intolerable for most cancer patients, and the prognosis is usually poor. Finally, cancer treatment costs are giant, and it is an unaffordable economic burden for ordinary people [5, 6]. Depression comorbidity reduces treatment compliance, weakens therapy outcomes, and increases cancer mortality. Therefore, antidepressant treatment is of importance and necessary for the cancer patient's comorbidity with depression [7] .

To make it worse, chronic stress might promote tumor growth. Our previous research showed that chronic stress promoted tumor growth in the mice model. [8, 9] As JUB has a variety of pharmacological activities, this work aims to investigate whether JUB exerts an antitumor effects in addition to its antidepressant effects, and a possible mechanisms based on tumor-bearing mice with depression-like phenotype induced by chronic unpredictable mild stress (CUMS).

## 2. Materials and Methods

### 2.1. Materials

Jujuboside B was purchased from Sigma Aldrich (St Louis, Mo, USA). The ELISA kits of 5-HT (PCDBA0104), IL-4 (PCDBM0168), IL-6 (PCDBM0170), IL-10 (PCDBM0143), and TNF-*α* (PCDBM0282) were from Immunoway Biotechnology Co., Ltd. (Beijing, China). p-MAPK, MAPK, p-ERK, ERK, p-Akt, Akt, p-PI3K, PI3K, p-CREB-1, Bax, and Bcl-2 antibodies were purchased from Abcam (USA).

### 2.2. Cells

Mice LLC lung cancer cells and human A549 cells were purchased from Shanghai Institutes of Cell Biology (Shanghai, China). Cells were cultured in the suggested DMEM culture medium containing 10% fetal bovine serum (Gibco), 100 *μ*g/mL streptomycin, and 100 U/mL penicillin at 5% CO_2_, 37°C.

### 2.3. Animals

C57BL/6 mice (8 weeks) were from Huachuang Sino Pharmaceutical Technology Co., Ltd. (number of qualitative qualifications: 2020032208). The mice were acclimated to the system for one week without any stress. Mice behaviors were assessed with the open-field test to screen out the abnormal behavior mice. The research-involved animals were conducted by the Animal Research Ethics Committee in Fengxian Hospital, Southern Medical University, Shanghai, China.

### 2.4. Open-Field Test (OFT)

The OFT focused on observing the animals' locomotion and exploratory behaviors. The mice should be familiarized with the testing environment for at least 3 h. Animals were placed into the box (50 × 50 × 40 cm) in the same position in turn, and the animal behavior analysis software was opened to spontaneously record the activities of the mice for 5 min. After each mouse was tested, 75% alcohol solution was used to avoid odor interference.

### 2.5. Sucrose Preference Test (SPT)

Followed by 12 h of 2% sucrose solution for adaption, then 18 h of water deprivation, mice were exposed to 2% sucrose solution and water for 2 h. The intake of liquid was recorded and calculated.

### 2.6. Animal Grouping

**Figure 1:**
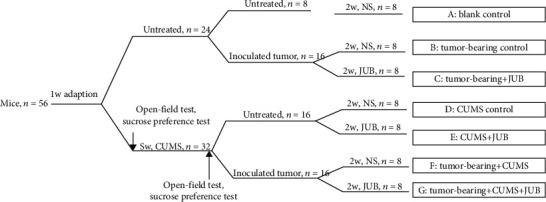
The flow chart of this whole study.

Fifty-six female mice were randomly divided into 7 groups (*n* = 8): A (blank control), B (tumor-bearing control), C (tumor-bearing + JUB), D (CUMS control), E (CUMS + JUB), F (tumor-bearing + CUMS), and G (tumor-bearing + CUMS + JUB). Seven groups of mice were given, respectively, different manipulations (Figure 1). Mice in CUMS-induced groups were fed separately and given nine different stressors randomly for 8 weeks (Table 1). To avoid prediction, the stressors were not repeated consecutively. 8 weeks later, to verify the CUMS model established successfully (groups D, E, F, and G), LLC cells (2 × 10^5^) were inoculated subcutaneously into the right flank of the animals (groups B, C, F, and G). After 3 days of cancer cell inoculation, JUB (40 mg/kg/day) was administrated intraperitoneally to mice (groups C, E, and G) for 2 weeks. Simultaneously, mice in group A, group B, group D, and group F were given vehicle intraperitoneally. Finally, all animals were executed, and serum and tumors were collected. Tumor weight and volume were measured.

### 2.7. RNA Sequencing

The total RNA of tumor tissue was isolated using the Trizol reagent following the protocol. Generated the cluster and sequenced libraries, 150 bp paired-end reads were collected. About 63 G reads and 430 M clean reads for all samples were collected. The clean read was calculated using cufflinks, and the read counts were obtained by HTSeq-count. *P* < 0.05 and fold change >2 or <0.5 were considered statistically significant.

### 2.8. Serum 5-HT, Trp, TNF-*α*, and IL-4, -6, and -10 Assay

The serum levels of 5-HT, Trp, TNF-*α*, IL-4, -6, and -10 in serum were measured by ELISA kits following the standard procedure.

### 2.9. mRNA Expression Study

Total RNA from tumor tissue was isolated with Trizol following the standard procedure. For RT-qPCR, RNA reverse transcription and PrimeScript RT-qPCR kit were performed according to the protocol (Takara, 9109, RR037A). The 7500 Real time Quantitative PCR systems were used to run PCR. Primer sequences were synthesized by Sangon Biotech (Table 2).

### 2.10. Protein Expression Analysis

Tumor tissue proteins were obtained with RIPA lysate (NCM Biotechnology, China). Then, the level of protein was detected by the BCA method (Yoche Biotechnology, China). Proteins were sequentially denatured, electrophoresed, and transferred to an NC membrane (Merk Millipore, Ireland). Which blocked with skimmed milk for 1 hour and incubated overnight at 4°C with diluted antibodies (p-MAPK, MAPK, p-ERK, ERK, p-Akt, Akt, p-PI3K, PI3K, p-CREB-1, Bax, and Bcl-2 were 1 : 1000; *β*-actin was 1 : 2000), incubated with secondary antibodies for 1 hour. Membranes were detected with ECL reagents (NCM Biotechnology, China).

### 2.11. Statistical Analysis

All values were expressed as means ± SD. Student's *t*-test was used in comparison between two groups, and one-way analysis of variance (ANOVA) (two-tailed) was used in the inter group. *P* value <0.05 was considered statistically significant.

## 3. Results

### 3.1. Behavioral Establishment of CUMS Model

**Figure 2:**
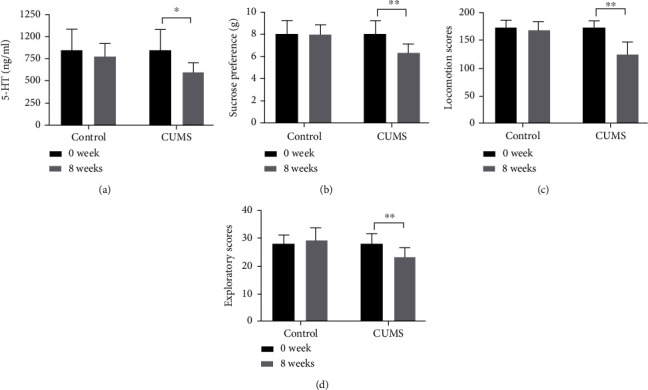
(a) 5-HT level, (b) sucrose preference, (c–d) locomotion scores, and exploratory scores in control and CUMS groups. Data are mean ± SD (*n* = 8/group). ^∗^P < 0.05 and^∗∗^P < 0.01.

After 8 weeks of model establishment, there was a striking difference in the behavioral and sucrose preference test between CUMS model mice and normal feeding mice. Compared with baseline, the behavioral scores (including locomotion score and exploratory score) and pleasure scores (sucrose preference) were decreased in CUMS group. Interestingly, there was no change in normal feeding animals (Figure 2). These data suggested that chronic stress successfully induced a depression-like phenotype.

### 3.2. JUB Ameliorated the Depression-Like Phenotype

**Figure 3:**
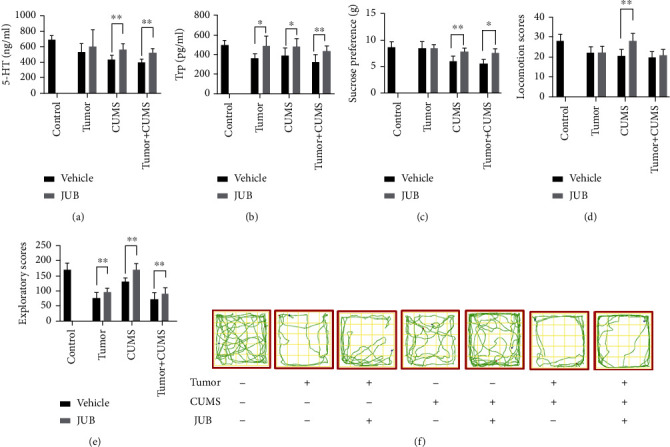
JUB ameliorated the depression-like behavior. Biochemical tests (a–b), sucrose preference (c), locomotion scores, and exploratory scores (d–e) before and after JUB (40 mg/kg/day) or vehicle administration for 2 weeks. (f) Movement of open-field test. Data are mean ± SD (*n* = 8/group). ^∗^P < 0.05 and^∗∗^P < 0.01.

In nontumor groups, the behavioral scores (including locomotion score and exploratory score), pleasure scores (sucrose preference), 5-HT, and Trp levels of mice in group E (CUMS + JUB) were increased compared with those in group D (CUMS control) after 2 weeks with JUB administration. Meanwhile, in the tumor group, the depression-like behaviors of mice in group G (tumor-bearing + CUMS + JUB) were ameliorated compared with mice in group F (tumor-bearing + CUMS) (Figure 3). Thus, JUB could ameliorate depression-like phenotype in tumor and nontumor groups.

### 3.3. CUMS-Promoted Tumor Progression; JUB Reversed This Effect

**Figure 4:**
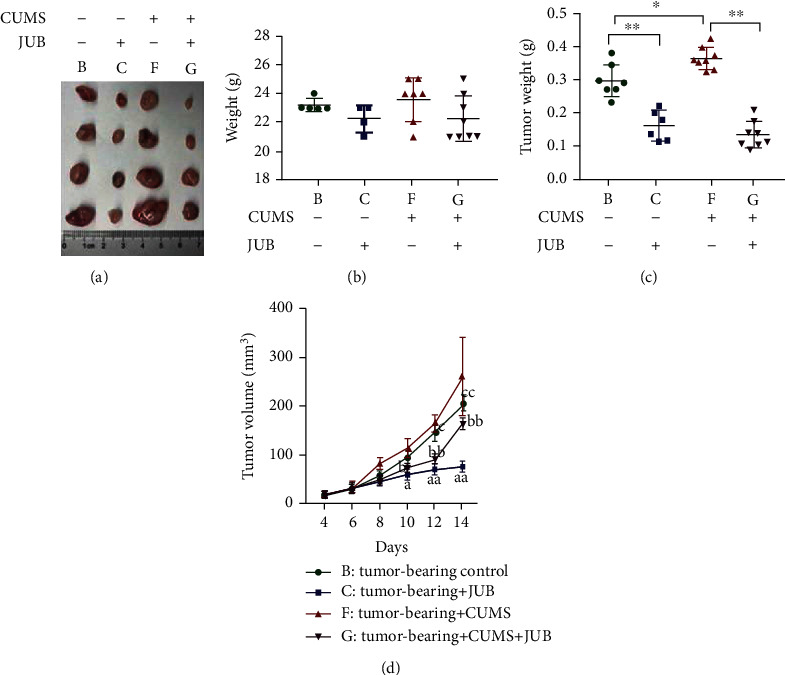
Effect of JUB and CUMS on tumor progression. (a) Observation of tumor tissues. (b) Tumor weight in different groups. (c) Mice body weight. (d) Volume of tumor. Data are mean ± SD (*n* = 8/group). ^∗^*P* < 0.05 and^∗∗^*P* < 0.01 ^*a*^*P* < 0.05 and ^*aa*^*P* < 0.01: tumor-bearing control vs. tumor-bearing + JUB; ^*b*^*P* < 0.05 and ^*bb*^*P* < 0.01: tumor-bearing + CUMS vs. tumor-bearing + CUMS + JUB; ^*c*^*P* < 0.05 and ^*cc*^*P* < 0.01: tumor-bearing control vs. tumor-bearing + CUMS.

Compared with group B (tumor-bearing control), the tumor volume in mice of group F (tumor-bearing + CUMS) was clearly bigger, suggesting chronic stress promoted the growth of the tumor. The tumor volume of group C (tumor-bearing + JUB) decreased significantly in comparison with that of group B (tumor-bearing control). Similarly, there was a significant decrease in tumor volume between mice in group G (tumor-bearing + CUMS + JUB) and group F (tumor-bearing + CUMS). The results suggested that JUB inhibited tumor progression and has an antitumor effect both in normal feeding tumor-bearing mice and in chronic stress-tumor-bearing mice (Figures 4(a) and 4(b)). In addition, CUMS and JUB did not affect the body weight in all groups (Figure 4(c)).

### 3.4. JUB Modulated Inflammatory Response

**Figure 5:**
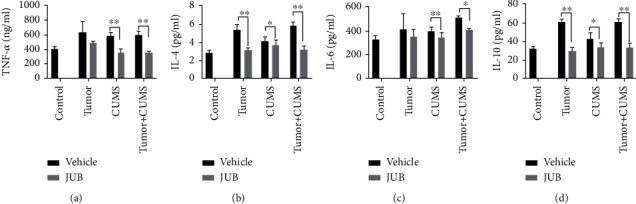
Expression of inflammatory cytokines of mice. (a–d) JUB decreased TNF-*α* and IL-4, -6, and -10 levels of LLC transplanted-bearing mice. Data are mean ± SD (*n* = 8/group). ^∗^*P* < 0.05 and^∗∗^*P* < 0.01.

Compared with group A (blank control), chronic stress significantly increased the inflammatory cytokines (TNF-*α* and IL-4, -6, and -10) in group D (CUMS control). Besides, JUB decreased TNF-*α* and IL-4, -6, and -10 levels in mice of groups C (tumor-bearing + JUB) and G (tumor-bearing + CUMS + JUB) as compared with the corresponding control groups B (tumor-bearing control) and F (tumor-bearing + CUMS) (Figure 5). These results indicated that JUB modulated the inflammatory response.

### 3.5. CUMS Activated CREB; JUB Dephosphorylated the Downstream Signaling Regulator CREB

**Figure 6:**
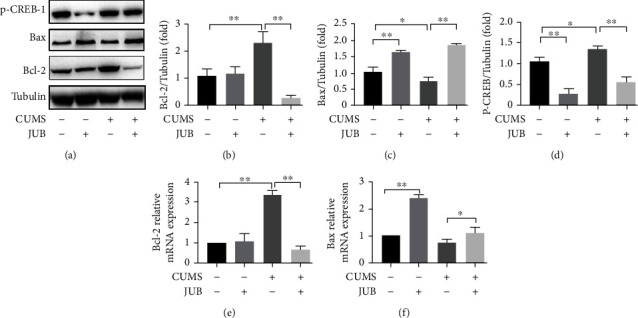
Effect of JUB and CUMS on inflammatory and apoptosis-related gene expression in the transplanted LLC tumors. (a) Protein expression bands of tumor tissues. (b–d) Statistical analysis of protein expression of p-CREB-1, Bcl-2, and Bax. (e–f) mRNA expression of transplanted LLC tumors. Data are mean ± SD (*n* = 8/group). ^∗^*P* < 0.05 and^∗∗^*P* < 0.01.

CREB is a protein involved in emotions, tumor cell apoptosis, and immune response. In the JUB group, p-CREB-1 was noticeably decreased in comparison with that in mice of group B (tumor-bearing control) and group F (tumor-bearing + CUMS), respectively. The protein and mRNA levels of proapoptotic Bax in mice of group C (tumor-bearing + JUB) and in mice of group G (tumor-bearing + CUMS + JUB) were significantly increased, while the protein and mRNA levels of antiapoptotic Bcl-2 was significantly decreased in comparison with group B (tumor-bearing control) and group F (tumor-bearing + CUMS). These results displayed that chronic stress enhanced the Bcl-2, p-CREB expression, and downregulated Bax expression (Figure 6); however, JUB administration reversed this phenomenon.

### 3.6. JUB Blocked the Signal Transduction Pathway of PI3K/Akt and MAPK/ERK

**Figure 7:**
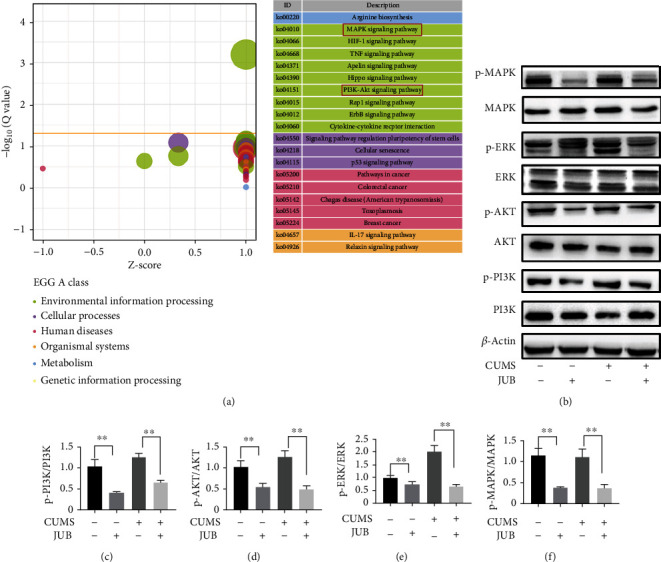
Effect of JUB and CUMS on apoptosis proteins in tumor tissues. (a) mRNA-seq of tumor in group B and F. (b) Protein expression bands of tumor tissues. (c–f) Statistical analysis of p-PI3K/PI3K, p-Akt/Akt and p-MAPK/MAPK, and p-ERK/ERK. Data were expressed as mean ± SD (*n* = 8/*g*roup). ^∗^*P* < 0.05 and^∗∗^*P* < 0.01.

Based on the existing literature, PI3K/Akt and MAPK/ERK are two crucial upstream cascades of activated CREB. In our study, CUMS activated the pathway of PI3K/Akt and MAPK/ERK. In group F (tumor-bearing + CUMS), PI3K-Akt and MAPK single pathways were activated compared with the tumor control group (Figure 7(a)). The p-PI3K, p-Akt, p-MAPK, and p-ERK protein expression in JUB groups was significantly decreased in comparison with the corresponding control group. Thus, CUMS enhanced the signal transduction pathway of PI3K/Akt and MAPK/ERK, but JUB blocked these signal transductions.

### 3.7. JUB Inhibits Tumor Progression In Vitro

**Figure 8:**
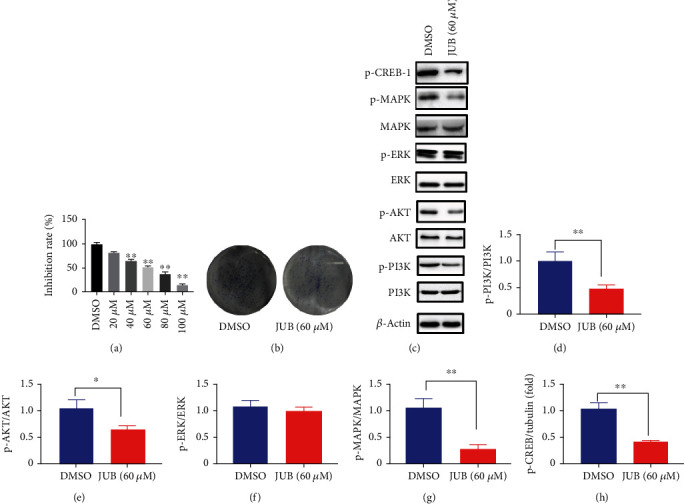
JUB inhibits tumor progression in A549 cells. (a) Cell growth-inhibition rate of JUB. (b) Clone formation. (c) Representative western blotting bands of A549 cells. (d–h) Statistical analysis of p-PI3K/PI3K, p-Akt/Akt and p-MAPK/MAPK, p-ERK/ERK, and p-CREB/Tubulin. Data were expressed as mean ± SD (n = 8/group). ^∗^*P* < 0.05 and^∗∗^*P* < 0.01.

The half maximal inhibitory concentration (IC50) of JUB was about 60 *μ*mol/L in A549 cells (Figure 8(a)). JUB reduced clonal formation in comparison with the control group (Figure 8(b)). Moreover, JUB blocked the signal transduction pathway of PI3K/Akt and MAPK/ERK, dephosphorylating the level of PI3K, Akt, MAPK, and CREB (Figures 8(c)–8(h)). The results in vivo were consistent with in vitro.

## 4. Discussion

For a decade, chronic stress or chronic stress-induced depression-like phenotype has been documented to promote cancer progression [10–12]. Chronic stress induces low-grade inflammation and impaired immune homeostasis [13–15], and finally promoted tumor growth [16, 17]. Clinical studies also revealed that chronic depression increased cancer occurrence and contributed to the development of malignant tumors [18]. Cancer patients with major depression were more possible correlated with cancer metastasis [10, 11, 19]. Generally, stressful life experiences are related to poorer cancer prognosis, lower survival, and higher mortality. Therefore, it is important to pay attention to depression comorbidities in cancer treatment.

Much evidence demonstrated that some antidepressants exerted antitumor effects [20–22]. For example, fluoxetine has been found to play a proactive role in antitumor progression in lung cancer and hepatocellular carcinoma [21]. Sertraline was able to regulate cancer multidrug resistance [20]. In our previous studies, we found that fluoxetine reverses multidrug resistance in breast cancer cells which might be medicated by inhibition of glutathione s-transferase-*π* and p-glycoprotein [4, 12]. And lately, we confirmed that the antidepressant drug fluoxetine exerts an antitumor effect via inhibiting enzymes related to the knurine pathway and enhancing T cellular immunity in NSCLC animals [8].

As traditional Chinese medicines usually have multiple pharmacological activities, in this work, we focused on Jujuboside B, the most effective component of the dried seed of *Zizyphi jujuba var. spinosi* (Bunge) Hu ex H.F. Chou [23]. We established the chronic stressed tumor-bearing comorbidity mice model successfully as confirmed by the behavioral scores (locomotion and exploratory scores) and sucrose preference test. The model was consistent with the core symptoms of anhedonia and social activity decline in patients with depression.

Our results suggested that chronic stress has a detrimental impact on oncotherapy, and JUB might have some reverse effects. On the one hand, the tumor in CUMS group mice was bigger than the tumor in non-CUMS group. JUB remarkably reversed the tumor-promoted compared with vehicle, in addition to its depression-like phenotype amelioration in chronic stress mice. Our work was consistent with research that JUB exerted anticancer effects in acute leukemia, gastric cancer, and colon cancer [1, 2, 15]. On the other hand, chronic stress or depression and inflammation usually fuel one another [24]. Chronic stress has a detrimental impact on immune system functions both in human beings and in animals [25]. In this study, chronic stress increased TNF-*α* and IL-4, -6, and -10 levels. JUB significantly reversed these upregulations, which implied that the antitumor effect of JUB may be related to the stress-immune-cancer axis.

As CREB regulated the gene expression, which is related to cancer cell growth [26–28], inflammation [29, 30], and emotion [31, 32], we measured the protein expression of CREB. We found that chronic stress increased p-CREB-1 expression significantly. Simultaneously, PI3K/Akt and MAPK/ERK, two crucial upstream cascades of activated CREB, were activated by CUMS, but JUB blocked this signal transduction pathway. The results were consistent with our cell experiment data.

Taken together, this work demonstrated that chronic stress promoted tumor growth. JUB exerts dual antidepressant and antitumor effects in tumor-depression comorbidity model mice. The effect might be related to blocking the signal transduction pathway of PI3K/Akt and MAPK/ERK and dephosphorylating the downstream signaling regulator CREB. For cancer patients with depression, both doctors and their families should pay more attention to them. JUB deserves further investigation as an adjunct to cancer treatment in the future.

## 5. Conclusions

**Figure 9:**
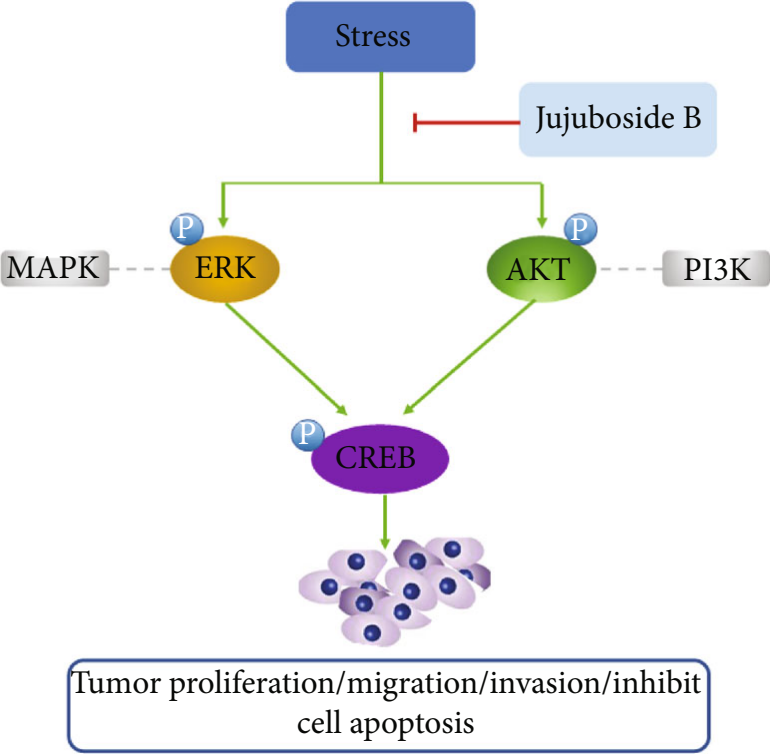
Working model of JUB-induced inflammatory response.

This study reveals that depression can promote the development of cancer; JUB exerts dual antidepressant and antitumor effects in tumor-depression comorbidity model mice. The antitumor effect of JUB on depression and tumor progression by blocking the signal transduction pathway of PI3K/Akt and MAPK/ERK and dephosphorylating the downstream signaling regulator CREB is shown in Figure 9. These findings also reminded us to pay attention to the treatment of cancer patients with depression and provide new perspectives on the molecular targets of JUB.

## Figures and Tables

**Table 1:** The stressors of CUMS.

Stressor	Duration
Clip tail	1 minutes
Restraint	1 hour
4°C water swimming	5 minutes
Noise	10 minutes
Humid environment	24 hours
Horizontal direction vibration	10 minutes
Day and night reversed	24 hours
Cage tilting	24 hours
45°C water swimming	5minute

**Table 2:** Primer sequences of mRNA analysis.

Gene name	Forward	Reverse
GAPDH	GACATGCCGCCTGGAGAAAC	AGCCCAGGATGCCCTTTAGT
Bax	TGCTGCCTTTTCTGTTCCTT	AAGGTGCTGGGTAGGGAAGT
Bcl-2	GTCCACGAACCCGTAAGGT	CATCTTTTCCCGATAGGTCCA

## Data Availability

The datasets used and/or analyzed during the current study are available from the corresponding author on reasonable request.
